# Design of an Antifungal Surface Embedding Liposomal Amphotericin B Through a Mussel Adhesive-Inspired Coating Strategy

**DOI:** 10.3389/fchem.2019.00431

**Published:** 2019-06-18

**Authors:** Diana Alves, Ana Teresa Vaz, Tânia Grainha, Célia F. Rodrigues, Maria Olívia Pereira

**Affiliations:** Laboratório de Investigação em Biofilmes Rosário Oliveira (LIBRO), Centre of Biological Engineering (CEB), University of Minho, Braga, Portugal

**Keywords:** antifungal coating, dopamine chemistry, catheter-associated urinary tract infections, liposomal amphotericin B, *Candida albicans*

## Abstract

Microbial colonization of urinary catheters remains a serious problem for medicine as it often leads to biofilm formation and infection. Among the approaches reported to deal with this problem, surfaces functionalization to render them with antimicrobial characteristics, comprises the most promising one. Most of these strategies, however, are designed to target bacterial biofilms, while fungal biofilms are much less taken into account. In real-life settings, fungi will be inevitably found in consortium with bacteria, especially in the field of biomaterials. The development of antifungal coating strategies to be combined with antibacterial approaches will be pivotal for the fight of biomaterial-associated infections. The main goal of the present study was, therefore, to engineer an effective strategy for the immobilization of liposomal amphotericin B (LAmB) on polydimethylsiloxane (PDMS) surfaces to prevent *Candida albicans* colonization. Immobilization was performed using a two-step mussel-inspired coating strategy, in which PDMS is first immersed in dopamine solution. Its polymerization results in the deposition of a thin adherent film, called polydopamine (pDA), which allowed the incorporation of LAmB, afterwards. Different concentrations of LAmB were screened in order to obtain a contact-killing surface with no release of LAmB. Surface characterization confirmed the polymerization of dopamine and further functionalization with LAmB yielded surfaces with less roughness and more hydrophilic features. The proposed coating strategy rendered the surfaces of PDMS with the ability to prevent the attachment of *C. albicans* and kill the adherent cells, without toxicity toward mammalian cells. Overall results showed that LAmB immobilization on a surface retained its antifungal activity and reduced toxicity, holding therefore a great potential to be applied for the design of urinary catheters. Since the sessile communities commonly found associated to these devices exhibit a polymicrobial nature, the next challenge will be to co-immobilize LAmB with antibacterial agents to prevent the establishment of catheter-associated urinary tract infections (CAUTI).

## Introduction

Every day, countless lives are saved thanks to the advances achieved in medical devices. Alongside with the benefits of their introduction on medical practice, there is the growing number of biomaterial-associated infections (Donlan, 2001; Busscher et al., 2012). This is particular true for the use of indwelling urinary catheters. According to the World Health Organization, urinary tract infections comprise the most common hospital-associated infections (Tenke et al., 2017) and it was reported that approximately 70–80% of these infections are mainly observed in patients with catheters (Zarb et al., 2012; Lo et al., 2014).

Urinary catheters are hollow and flexible tubes functioning as a closed sterile system introduced through the urethra and kept in place by an inflatable balloon, which allows the urinary drainage from the bladder into an attached bag (Jacobsen et al., 2008). These devices allow, therefore, the introduction of opportunistic microorganisms providing, at the same time, an ideal surface for microbial colonization and subsequent biofilm formation (Tenke et al., 2017). Once inserted, the surface of catheters is altered by the formation of a conditioning film which results from the deposition of urine components (proteins, minerals, and polysaccharides). Microorganisms are then transported to the surface for their reversible adhesion through non-specific interactions including van der Waals, hydrophobic interactions, and electrostatic forces (Donlan and Costerton, 2002). Flagella and pili are also well-known virulence factors expressed by uropathogenic microorganisms which assist their attachment to catheter and epithelial cells of urinary tract (Flores-Mireles et al., 2015). In the next step, microorganisms become strongly attached to the surface and cell-surface association can occur as single cells or as clusters, forming a monolayer or multilayer biofilm, respectively. Attached microorganisms start to produce extracellular polymeric substances (EPS) such as polysaccharides, proteins, and extracellular DNA. EPS are essential for biofilm maturation and differentiation, since they are responsible for irreversible attachment and the development of three-dimensional structure of mature biofilms. Biofilm matrix also provide mechanical support and mediates the interactions cell-cell and cell-surface. Biofilm encasing microorganisms may eventually return to the planktonic life-style through dispersal and detachment of biofilm from the catheter (Flemming and Wingender, 2010).

Biofilm formation is one of the main challenges faced by urinary catheters, making CAUTI extremely difficult to treat (Azevedo et al., 2017). Microorganisms in this sessile community are more resistant to antimicrobials and the host immunity. The best approach to fight CAUTI relies on preventing the establishment of biofilms and it can be accomplished by modification of urinary catheter surfaces so they can resist microbial colonization (Yu et al., 2017). A number of coating strategies have been reported with this aim, but most of them comprise mechanisms to prevent the colonization of bacteria (Lim et al., 2015; Cooper et al., 2016; Singha et al., 2017). As stated by the European Center for Disease Prevention and Control, in an annual epidemiological report from 2014, the most commonly isolated microorganisms from catheter infections were *Escherichia coli* (28%) followed by *Candida* species (18%). Furthermore, it has been demonstrated that a great number of catheters are colonized by three or more microorganisms and that only 12.5% of the infections are monomicrobial (Holá et al., 2010; Selek et al., 2016). Coating strategies targeting fungi species are, therefore, in great demand.

Amphotericin B is the most common polyene antifungal agent applied in medicine to treat a broad range of fungal infections (Pierce et al., 2013). Its mode of action involves the binding to ergosterol in the fungal cell membranes, causing ion leakage and subsequent cell death (Baginski and Czub, 2009). Amphotericin B deoxycholate (AmB), the first formulation developed, has been associated to some drawbacks, namely nephrotoxicity, low solubility and bioavailability (<0.9 %) (Anderson et al., 2017). In the last few years, efforts have been made, therefore, in the engineering of new formulations which are mainly based on lipids, colloidal suspension, and polymers (Saldanha et al., 2016; Tan et al., 2016; Shu et al., 2018). Among these formulations, liposomal amphotericin B (LAmB) has been used in the last two decades since it has been demonstrated to have less toxicity as compared to Amphotericin B deoxycholate. This formulation has also exhibited a better performance in the treatment of biofilm cells of different *Candida* spp. as compared to Amphotericin B deoxycholate (Rodrigues and Henriques, 2017).

The aim of the present study was to immobilize LAmB on PDMS, a material commonly used for urinary catheters manufacture, to impart it with the ability to resist *Candida albicans* colonization. A mussel-inspired coating strategy was explored for its immobilization so that LAmB retained its antimicrobial activity without significant leaching from the surfaces, to overcome toxicity issues associated and the potential for development of microbial resistance.

## Materials and Methods

### Organism and Growth Conditions

The reference strain *C. albicans* SC 5314 was used in this study. After being streaked on a Sabouraud dextrose agar (SDA, Liofichelm) plate, from a frozen stock solution, this strain was grown for 24 h at 37°C. For each assay, some colonies were collected from the SDA plates and grown overnight in batches of Sabouraud dextrose broth (SDB, Liofichelm) at 37°C and 120 rpm. Cells were then harvested by centrifugation (3,000 g, 10 min, 4°C) and washed twice with phosphate buffered saline (PBS, pH = 7.5). Cellular density was then adjusted using a Neubauer counting chamber.

### Antifungals

Amphotericin deoxycholate (AmBdeox) was purchased from Sigma (Sigma-Aldrich, USA) and Liposomal Amphotericin B (LAmB) was supplied by Gilead^®^ (Foster City, CA, USA). Aliquots of AmBdeox were prepared using DMSO according to the indications of the manufacturer while the aliquots of LAmB were prepared in 10 mM bicine buffer.

### PDMS Preparation

PDMS (kit Sylgard 184, Dow Corning, USA) was prepared as recommended by the manufacturer's instructions. In summary, base and curing agents provided in the kit were mixed in 10:1 (w/w), casted in a petri dish and kept at room temperature for 48 h. Afterwards, PDMS was cut into circle pieces with 0.9 cm diameter and a thickness of ~0.3 cm. Before utilization, surfaces were subjected to an ultrasonic cleaning treatment to remove impurities and grease, in a commercial detergent (Sonasol, Henkel Ibérica, Portugal) for 5 min followed by methanol for 20 min and finally rinsed with distilled water. Cleaned coupons were sterilized at 121°C for 15 min, in autoclave.

### Polydopamine Coating and Further Immobilization of LAmB

**Figure 1:**
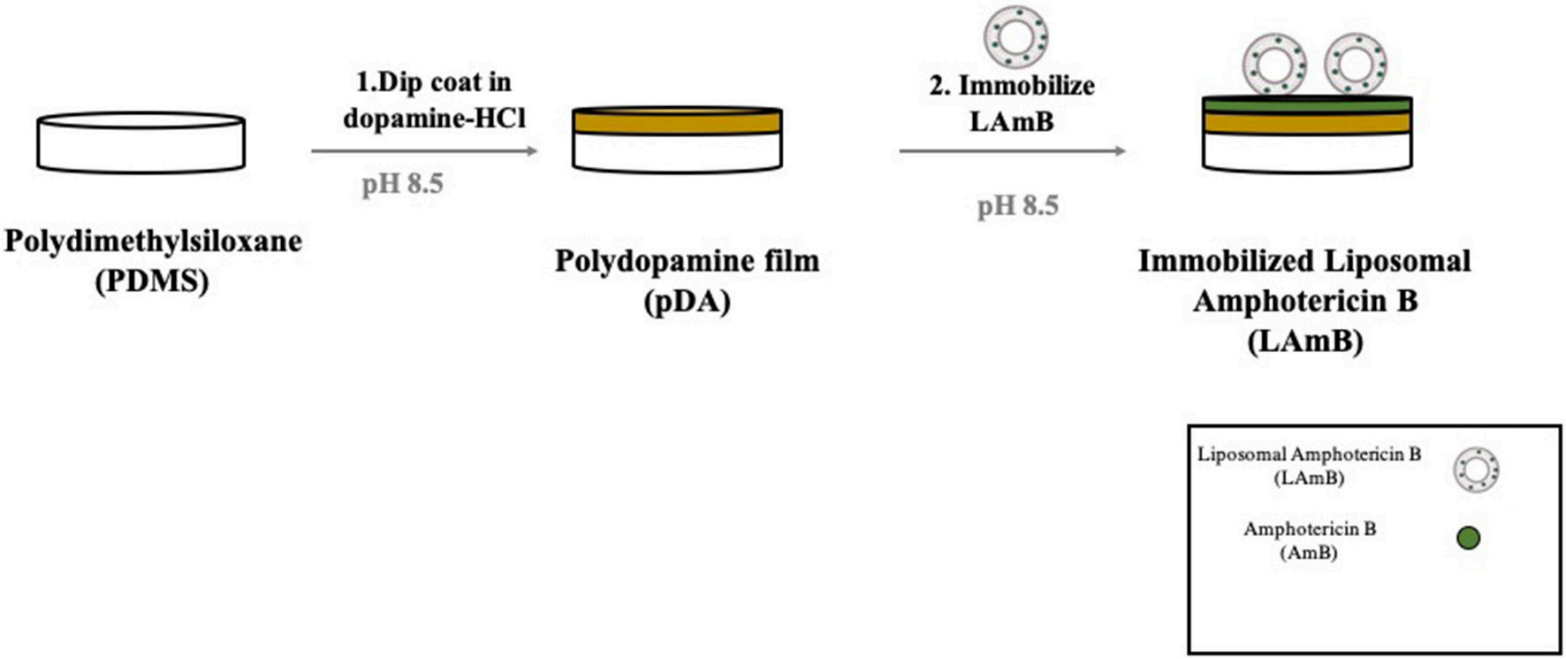
Schematic representation of polydopamine (pDA) coating developed for liposomal Amphotericin B (LAmB) immobilization on polydimethylsiloxane (PDMS). Materials were first functionalized with a layer of pDA, followed by LAmB immobilization adapted from Xu et al. (2017).

Liposomal amphotericin B immobilization was performed using a two-step mussel-inspired coating strategy previously reported (Lee et al., 2007) and it is schematically presented in Figure 1. The first step comprises the formation of a pDA layer which was accomplished by immersing the coupons in 7 mL of a freshly prepared solution of dopamine (Sigma, 2 mg/mL) in 10 mM bicine buffer (Sigma, pH 8.5) for 18 h at room temperature under agitation (70 rpm). Surfaces were then rinsed with ultrapure water. For LAmB immobilization, pDA coated coupons were immersed on a solution of LAmB prepared at different concentrations (0.5, 1, and 2 mg/mL) in bicine buffer, and they were incubated overnight at room temperature, under agitation (70 rpm). Functionalized surfaces were washed with ultrapure water and dried prior utilization.

### Fungi Contact-Killing and Leaching Assays

The contact-killing properties of PDMS functionalized with LAmB were evaluated as previously reported (Alves and Pereira, 2016). Briefly, a yeast suspension was adjusted in SDB to a final concentration of approximately 10^6^ colony-forming units (CFU) CFU/mL, and 20 μL of this solution was added on top of PDMS coupons placed in a petri dish. The plate was afterwards sealed and incubated at 37°C under static conditions for 24 h. After contact, coupons were placed on a SDA plate, with the face exposed to the yeast suspension, in contact with the agar, and they were incubated for additional 24 h at 37°C. Yeast growth was evaluated for the conditions tested and tabulated as “+” for growth and “–” for no visible growth.

To evaluate the possible leaching of LAmB from the PDMS modified surfaces, an analytical method previously reported was adapted (Asri et al., 2014). Surfaces were placed on top of an agar plate previously streaked with *C. albicans* adjusted to a concentration of approximately 10^8^ CFU/mL. Plates were then incubated for 72 h at 37°C and the presence or absence of an inhibition zone was evaluated. The inhibition zone as an indication of LAmB leaching from the surfaces. Three independent assays with three replicates for each condition were performed.

### Surface Characterization

#### Surface Morphology, Chemical Composition, and Roughness

The surface morphology of materials was analyzed by scanning electron microscopy (SEM). Prior to observation, samples were covered with a very thin film of Au-Pd (80–20 weight%), 5 nm, in a high resolution sputter coater and observed with an Ultra-high resolution Field Emission Gun Scanning Electron Microscopy (FEG-SEM), NOVA 200 Nano SEM, FEI Company. Topographic images were performed with a secondary electron detector at an acceleration voltage of 10 kV. Chemical composition of the surfaces was also investigated through the Energy Dispersive Spectroscopy (EDS), using an EDAX Si (Li) detector, with an acceleration voltage of 15 kV. Surface roughness was evaluated using atomic force microscopy (AFM). AFM measurements were performed at room temperature using a Nanoscope III Multimode Atomic Force Microscope (Digital Instruments) operating in tapping mode. Scan rates were set at 1 Hz and the scanning area per sample was fixed at 5 ×5 μm.

#### Surface Hydrophobicity Parameters

Hydrophobicity parameters of material surfaces were established using the sessile drop contact angle method. Contact angles were measured on an automated contact angle device (OCA 15 Plus, Dataphysics, Germany) provided with image acquisition and data analysis. Measurements were performed at room temperature, using 3 μL drops of reference liquids for standardized contact angles measurements: water, glycerol and α-bromonaphtalene. The van Oss approach (Van Oss and Giese, 1995) was then applied to associate the contact angles with the hydrophobicity parameters.

### Fungi Viability on Modified Surfaces

The performance of modified surfaces against yeast adhesion was evaluated by preparing a suspension with approximately 10^8^ CFU/mL in artificial urine (Brooks and Keevil, 1997) to better mimic the environment of CAUTI. PDMS coupons, before and after modifications were placed on 48-well microtiter plates (Orange Scientific, USA), which were covered with 300 μL of yeast suspension and plates were incubated for 4 h, at 37°C and 120 rpm. Samples were then washed with saline solution (0.9% NaCl), stained with a live/dead stain (BacLight Bacterial Viability Kit, Invitrogen) and observed in a fluorescent inverted microscope (Olympus, BX51). The staining solution is composed by propidium iodide and the SYTO 9, two nucleic acid staining agents, which allow the differentiation between viable and non-viable cells, evaluating membrane's integrity. Propidium iodide is a red-fluorescent nuclei acid staining agent which only penetrates damaged cell membranes while SYTO 9 is a green-fluorescent nucleic acid staining agent, which can penetrate cells both with intact and damaged membranes. At least three images per coupon were collected and at least three coupons were inspected per assay.

### Evaluation of Biofilm Formation by CFU Counting

The potential of the modified surfaces to impair biofilm formation, was investigated by enumerating the number of yeast cells adhered to the surfaces. A yeast suspension was first prepared from an overnight culture and adjusted to a final concentration of approximately 10^8^ CFU/mL in artificial urine. The coupons were placed in a 48-well microtiter plates and 300 μL of this suspension was added to each well. Plates were incubated at 37°C, under agitation (120 rpm) and cells were allowed to attach to the surfaces for 1 h. Coupons were then washed twice with saline solution and placed in new wells of a microtiter plate and 300 μL of artificial urine were then added to each well. Plates were then incubated for more 23 h under the same conditions. After this period, coupons were washed again with saline solution to remove free-floating yeast, transferred to new wells and 300 μL of PBS were finally added to each well and the surfaces were scraped. Solutions resulting from the scraping were collected, vortexed to disrupt possible cell aggregates. Serial 10-fold dilutions were performed and plated into SDA plates which were incubated overnight at 37°C in an aerobic incubator prior enumeration. To infer about pDA role on LAmB immobilization, the sample PMDS-LAmB was also evaluated in this assay. This one was prepared by simple immersion of PDMS coupons on a solution of LAmB (2 mg/mL) using the same conditions of pDA-LAmB (overnight at 70 rpm and room temperature).

### Cytotoxicity Assay

Cytotoxicity evaluation was performed according to the ISO 10993-5:2006, using fibroblast cells 3T3 (CCL 163) obtained from ATCC, commonly used for biomaterial surface biocompatibility studies (Lee et al., 2004a; Zhu et al., 2006). Dulbecco's modified Eagle's medium (DMEM) supplemented with 10% of fetal bovine serum and 1% penicillin/streptomycin was used to culture these cells at 37°C, 5% CO_2_. Once achieved the confluence, trypsin was used to detach cells and a cell suspension was prepared with 10^5^ cells/mL, which was then added to each well of a 48-well microtiter plate, in which the PDMS surfaces were previously inserted. Plates were incubated at 37°C, 5% CO2 for 48 h and after that period of time, cytotoxicity was evaluated by the MTS (3-(4,5-dimethylthiazol-2-yl)-5-(3-carboxymethoxyphenyl)-2-(4-sulfophenyl)-2H-tetrazolium) inner salt reduction assay as a measure of cellular metabolic viability. For that, medium was removed and a solution containing 100 μL of MTS (Promega CellTiter 96^®^ AQueous Non-Radioactive Cell Proliferation Assay) per each 1 mL of DMEM without phenol red was added to each well. After 1 h of incubation in the dark, at 37°C, 5% CO2, the absorbance of the resulting solution was red at 490 nm and results were expressed as percentage of viable cells using the metabolic activity of cells grown on pDA-coated PDMS as controls.

### Statistical Analysis

Results were presented as mean ± standard deviation (SD). Statistical analysis was performed by Kolmogorov-Smirnov normality test using Graph Pad Prism 7.0 for Mackintosh. Afterwards, parametric tests (one-way Anova followed by Tukey's test) or non-parametric (Kruskal-Wallis test) were implemented, depending on whether the samples were from normally distributed populations or not, respectively.

## Results and Discussion

The drawbacks arising from bacterial colonization of indwelling devices such as urinary catheters have been well-established, as evidenced by a massive body research on antibacterial coating strategies. More recently, it has become clear that fungal species attach to these devices, either by themselves but mainly together with bacteria, playing a crucial role on the pathogenesis of poly-kingdom biofilm infections (Costa-Orlandi et al., 2017). The main goal of the present study was, therefore, to propose an antifungal coating strategy to prevent the colonization of *C. albicans*, the most common fungus associated with indwelling medical device infections (Mukherjee et al., 2009). PDMS, commonly known as silicone rubber, was employed as the model surface for functionalization, to better mimic the surface chemistry of silicone urinary catheters (Lee et al., 2004b). Surface modification was performed using a simple, versatile, and cost-effective approach, based on dopamine chemistry, previously applied for the immobilization of several antibacterial agents (Sileika et al., 2011; Alves and Pereira, 2016; Alves et al., 2016). To provide the antifungal features, a lipid formulation of Amphotericin B (liposomal amphotericin B) was chosen. The mechanism of action proposed for this formulation relies on the binding of liposomes to cell walls and subsequent dissociation of free amphotericin B to cross by itself through the cell wall until reach the cell membrane, promoting the leakage of intracellular components and subsequent fungal cell death (Stone et al., 2016). Recently, it has been shown that liposomes are able to travel intact through the cell wall and release AmB only when they reach the inner side of the cell wall at the surface of the hydrophobic membrane. When not in contact with a fungus, AmB remains associated with the liposome layer, which explains its reduced mammalian cell toxicity when administered for the treatment of systemic fungal infections (Walker et al., 2018). Circulating liposomes can be rapidly cleared from the organism, so smaller amounts will reach the target site (Pasquardini et al., 2008). Immobilization on a surface comprises a promising strategy to overcome these issues.

### Immobilization of LAmB on PDMS Surfaces

For LAmB immobilization on PDMS surfaces, a two-step bio-inspired approach was applied (Figure 1). The first step comprised the deposition of an adhesive pDA “primer” coating on PDMS surfaces from a mildly basic solution of dopamine. The thickness of this layer formed in similar conditions has been previously measured by ellipsometry and it was of approximately 50 nm (Lee et al., 2007; Zhou et al., 2014). This parameter should not be significantly changed after LAmB immobilization as it has been demonstrated for other compounds, such as antimicrobial peptides (Cui et al., 2014). The presence of quinone functional groups in pDA coating allows further covalent immobilization of nucleophilic biomolecules via Michael addition and/or Schiff base reactions (Liebscher et al., 2013). Free Amphotericin B contains primary amines, so it was hypothesized that if some AmB was released from the liposomes during functionalization of pDA-coated surface, this one could be grafted onto the surfaces through Michael addition and/or Schiff base reactions. As far as liposomes as concerned, since they do not present any amine or thiol groups in their composition, it was intended to promote its adsorption, taking advantage of the increased roughness provided by pDA-based coatings previously reported (Alves et al., 2016). It has been shown that low roughness promotes liposome rupture, but high roughness induces adsorption of whole liposomes (Duarte et al., 2015). Different concentrations of LAmB were tested in this second step. Since it was intended to provide PDMS with the ability to contact-kill fungal cells but without active AmB release, to avoid cytotoxic issues, optimization parameters in this phase included the following factors: contact-killing and leaching activities (Table 1).

**Table 1:** Contact-killing activity and leaching potential of PDMS functionalized with LAmB with different concentrations.

**LAmB concentration (mg/mL)**	**Fungal growth**	**Inhibition zone**
0.5	+	A
1	+	A
2	–	A

*Visible growth was used as an indication of contact killing activity and it was tabulated as “+” for fungal growth and “–” for no growth. Leaching of LAmB was evaluated by the presence (P) or absence (A) of an inhibition zone*.

Results showed that no contact-killing was observed for bare PDMS, before or after pDA coating (Supplementary Figure 1). Further functionalization with LAmB yielded the surfaces with fungal contact-killing activity only for the higher concentration tested (2 mg/mL), as evidenced by the absence of fungal growth in these modified coupons. Regarding the release of LAmB, for all the concentrations tested, no inhibition zone was found which was an evidence of no release from the surfaces or from the liposomes. If some AmB was released from the liposomes during immobilization, these results suggest that it was covalently bounded trough their amine groups via Michael addition and/or Schiff base reactions. Additionally, for comparison purposes, free AmBdeox was also immobilized, using the higher concentration of LAmB tested, and it was possible to observe an inhibition zone surrounding the functionalized coupons (Supplementary Figure 2), an evidence of AmB leaching, which highlight the role of liposomes on protecting AmB also when they are immobilized.

The overall results from these optimization tests allowed to determine the best concentration for the immobilization of LAmB to provide the surfaces of PDMS with antifungal activity but with no LAmB release from the surfaces (2 mg/mL). This coating strategy was further evaluated.

### Surface Characterization

**Figure 2:**
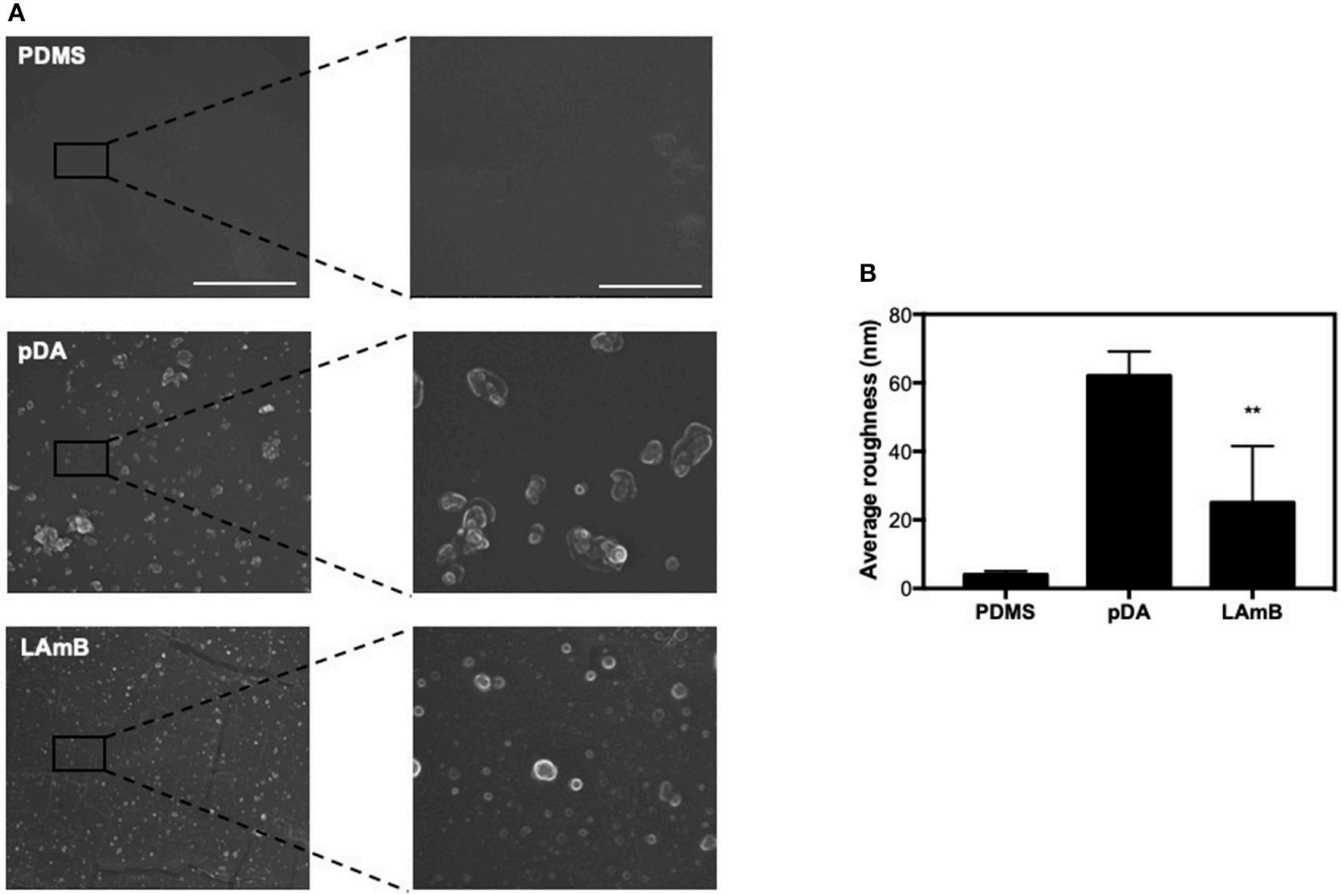
Surface characterization. SEM images **(A)** and surface average roughness **(B)** of unmodified polydimethylsiloxane (PDMS), and pDA before and after functionalization with LAmB. The scale bars in the left and right column indicate 10 and 2 μm, respectively. Significant differences were found for ^**^p < 0.01 compared to PDMS control surfaces.

Surface morphology after each step of functionalization was inspected by SEM analysis (Figure 2A). It was possible to observe that bare PDMS exhibits a smooth surface as compared to further modified surfaces. After pDA coating, surfaces revealed some aggregates resulting from self-polymerization of dopamine previously described (Alves et al., 2016). Further functionalization with LAmB did not introduce significant changes on this morphology. Since dopamine agglomerates are in the same range of liposomes loaded with AmB, it was not possible to discriminate them. For further surface morphology characterization, samples were also evaluated by AFM and from the images obtained, it was possible to determine the average roughness of surfaces (Figure 2B). Results corroborated that pDA coating increased the surface roughness, which may be explained by the presence of polydopamine agglomerates and it is in agreement with other studies (Xu et al., 2017). The additional layer of LAmB decreased the surface roughness as compared to pDA coating. Chemical analyses were also conducted using EDS and results are presented in Table 2. Results showed that PDMS before and after pDA coating displayed a similar chemical signature, which may be attributed to the sampling depth achieved by the EDS, which is higher than 50 nm (the maximal thickness of pDA coatings). Therefore, it is expected that PDMS is the major contributor to the chemical signature detected by EDS analysis. On the other hand, pDA-coated PDMS further functionalization with LAmB, resulted in chemical changes, namely an increase in carbon (C) content and decrease on silicon (Si) content, which is an evidence of LAmB successful coating.

**Table 2:** EDS quantification of atomic compositions on the polydimethylsiloxane surface (PDMS), pDA-coated PDMS (pDA), and pDA-coated PDMS surfaces functionalized with LAmB (LAmB).

**Sample**	**C (%)**	**O (%)**	**Si (%)**
PDMS	27.74	22.34	49.92
pDA	28.79	22.18	49.03
LAmB	35.28	21.03	43.68

The hydrophobicity parameters of surfaces were also determined using van Oss approach (Van Oss and Giese, 1995). Contact angles, surface tension parameters and free energy of interaction are summarized in Table 3. Results showed that bare PDMS presented a water contact angle higher than 65° and a negative value of free energy of interaction (Δ*G*${}_{\text{iwi}}^{\text{TOT}}$), which reveals the hydrophobic features of this material (Vogler, 1998). Application of a pDA coating, introduced hydrophilic features to the modified surfaces, as evidenced by the positive value of free energy of interaction and a lower water contact angle of 77.8°. This surface modification is attributed to dopamine polymerization that introduces new hydrophilic functional groups, especially the catechol and amine groups, on the PDMS surface. Further functionalization with LAmB increased the value of free energy of interaction which was attributed to the successful decoration with liposomes on the pDA-coated PDMS surfaces.

**Table 3:** Values of contact angles (°) with water (θ_W_), glycerol (θ_G_), α-bromonaphtalene (θ_B_), surface tension parameters (mJ/m^2^), and free energy of interaction (${\Delta G}_{iwi}^{TOT}$) (mJ/m^2^) between the surfaces (i) when immersed in water (w).

**Surface**	**Contact angle (**^****°****^**)**			**Surface tension parameters (mJ/m**^****2****^**)**			**Free energy of interaction (mJ/m^**2**^)**
	**θ_W_**	**θ_G_**	**θ_B_**	**$\gamma_{\text{i}}^{\text{LW}}$**	**$\gamma_{i}^{+}$**	**$\gamma_{i}^{-}$**	**$\Delta G_{iwi}^{TOT}$**
PDMS	112.1 ± 0.4	108.1 ± 1.4	51.2^*^± 1.3	26.4	0	5	−61.0
pDA	77.8^*^± 11.7	96.2^*^± 5.1	40.4^*^± 3.6	34.5	0	61.2	48.9
LAmB	69.9^*^± 7.3	91.0^*^± 1.6	36.1^*^± 1.2	36.3	0	71.4	61.1

Values are means ± SD. Significant differences were found for

* *p <0.05 compared to PDMS*.

### Antifungal Performance of Functional Coatings

**Figure 3:**
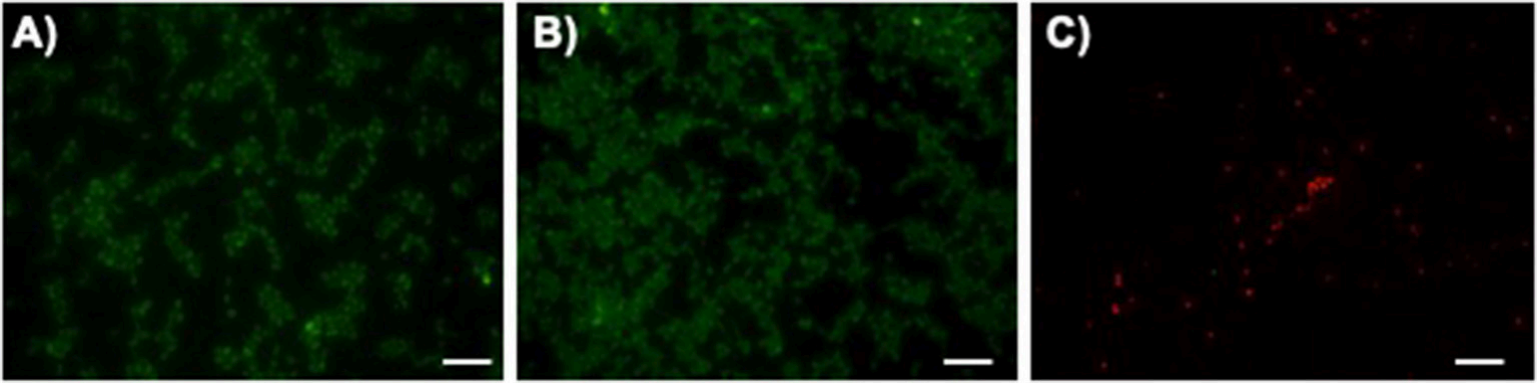
Fungal initial adhesion to surfaces. Representative fluorescent live/dead stain images obtained after adhesion of *C. albicans* for 4 h on unmodified polydimethylsiloxane (PDMS, **(A)**, pDA-coated PDMS surfaces (pDA, **(B)** and pDA-coated surfaces functionalized with LAmB (LAmB, **(C)**. The scale bar indicates 20 μm.

After confirming that LAmB retained its activity after immobilization, its antifungal performance was further evaluated. To inspect the ability of modified surfaces to interfere with *C. albicans* adhesion and/or viability, fungal cells were allowed to adhere for 4 h and the remaining cells on the PDMS surfaces were imaged with fluorescence microscopy (Figure 3). It has been established that the first 6 h are the critical time for preventing pathogens adhesion to the surfaces of biomaterial implants (Poelstra et al., 2002). It was possible to observe that fungal cells were able to adhere to both unmodified PMDS and pDA-coated surfaces without compromising their viability. Further immobilization with LAmB, on the other hand, provided PDMS surfaces with the ability to prevent fungal adhesion and promote cellular membrane disintegration, as evidenced by the lower number of attached cells and their red fluorescence color. The decreased number of adhered cells found on these surfaces is an evidence of their anti-adhesive features which may be attributed to the increased hydrophilicity and lower roughness, as compared to pDA coating alone (Figure 2 and Table 3). Contact-killing features observed may be attributed to AmB released from the liposomes during immobilization and subsequently grafted onto pDA coating via Michael addition and/or Schiff base reactions as well as to the immobilized liposomes that, upon contact with fungi will allow AmB release to target these cells membrane (Walker et al., 2018).

**Figure 4:**
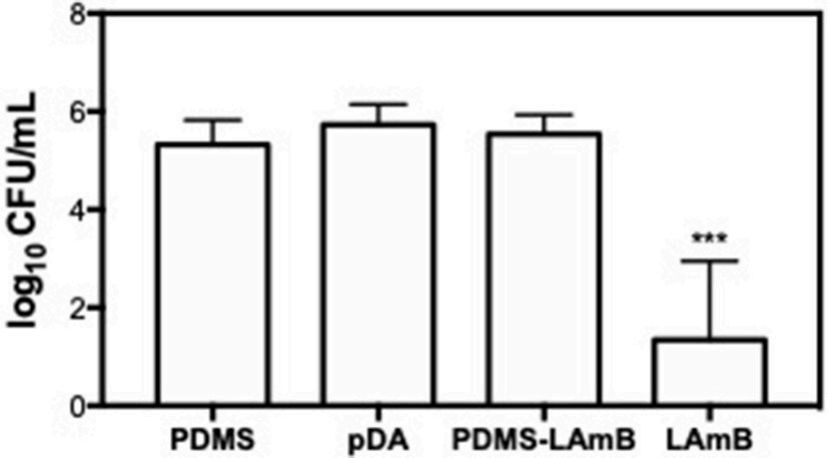
Number of adhered *C. albicans* cells after exposure to bare polydimethylsiloxane (PDMS), pDA-coated PDMS surfaces (pDA), PDMS after adsorption of LAmB (PDMS-LAmB) and pDA surfaces functionalized with LAmB (Lamb) for 1 h, followed by 23 h incubation in artificial urine. Significant differences were found for ^***^p < 0.001 compared to PDMS control samples.

The antifungal activity of LAmB was also investigated for a longer period of time: fungal colonization was allowed to proceed for 1 h, being afterwards exposed to urine for additional 23 h, to better mimic the physiological conditions. The number of fungal cells adhered to surfaces was enumerated by CFU counting (Figure 4). Results showed that PDMS with and without pDA coating favored the attachment of *C. albicans* as demonstrated for a shorter period of time. LAmB immobilized was able to impair fungal attachment as evidenced by an approximately 3 Log CFU reduction. To better understand the role of pDA layer on LAmB functionalization, physical adsorption of LAmB was allowed to proceed onto PDMS surfaces, and their antifungal properties were evaluated. Results showed that simple adsorption of LAmB did not impart the surfaces of PDMS with antifungal features, as *C. albicans* was able to adhere to these surfaces at the same extent as it did to PDMS and pDA controls. Such results are an evidence of pDA role for a stable functionalization of PDMS with LAmB.

### Biocompatibility of LAmB Immobilized

**Figure 5:**
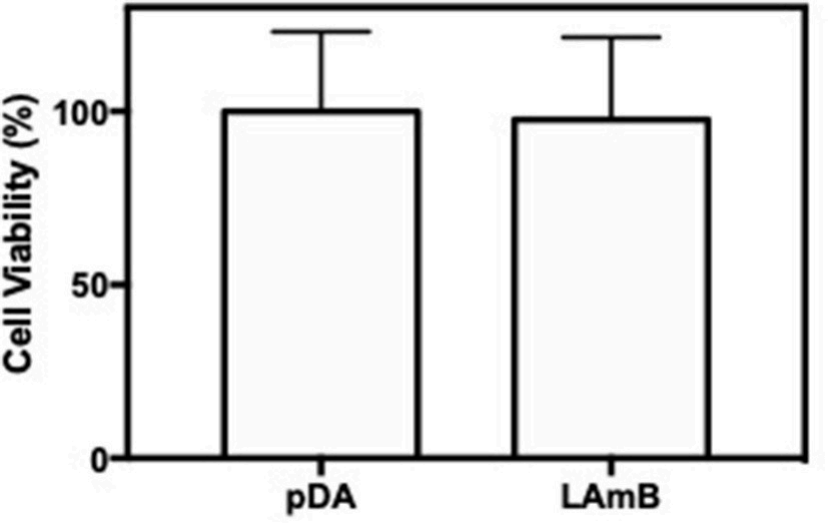
Surfaces biocompatibility. Viability of 3T3 fibroblast cells after 48 h of contact with pDA-coated PDMS surfaces before and after functionalization with LAmB, measured with an MTS assay. No significant differences were found.

An important advantage associated to the use of LAmB, is its reduced toxicity provided by their incorporation within the liposomes (Stone et al., 2016). Toxicity of LAmB, after functionalization, on mammalian cells was then evaluated. Results presented in Figure 5 showed that surfaces functionalized with LAmB did not compromise 3T3 fibroblast metabolic activity, as compared to pDA coating, which is an evidence of no toxicity. This ladder evidence also suggests no release of active AmB, which may be attributed to its entrapment on liposomes or its covalently attachment to the surface.

## Conclusions

The importance of fungal co-existence with bacteria in the pathogenesis of biomaterial-associated infections has only recently started to be recognized. Given the differences on the cell walls of bacteria and fungi, most of antibacterial coating strategies will not be antifungal as well. In this study a mussel-inspired coating strategy was successfully applied for the immobilization of LAmB. Although surface characterization showed that pDA coating imparted the surfaces of PDMS with hydrophilic features but increased roughness, the latter was then attenuated after LAmB functionalization. This approach also provided PDMS surfaces with remarkable antifungal and biocompatible features, holding, therefore great potential to be combined with antibacterial agents in the development of catheters able to prevent CAUTI.

## Data Availability

The raw data supporting the conclusions of this manuscript will be made available by the authors, without undue reservation, to any qualified researcher.

## Author Contributions

DA and MP designed the experiments. AV, DA, and TG carried out the experiments. DA, AV, CR, and MP analyzed data. DA wrote the manuscript. All authors approved the submitted manuscript.

### Conflict of Interest Statement

The authors declare that the research was conducted in the absence of any commercial or financial relationships that could be construed as a potential conflict of interest.
