# Supplementary material for: Design of an Antifungal Surface Embedding Liposomal Amphotericin B Through a Mussel Adhesive-Inspired Coating Strategy
Source: Front Chem. 2019 Jun 18;7:431. doi: 10.3389/fchem.2019.00431 (PMC6591271; doi:10.3389/fchem.2019.00431)
Supplement: Supplementary file 1 [file Table_1.DOCX]

Design of an antifungal surface embedding liposomal amphotericin B through a mussel adhesive-inspired coating strategy

Diana Alves^*^, Ana Teresa Vaz, Tânia Grainha, Célia F Rodrigues, Maria Olívia Pereira

*Correspondence: Diana Alves: [dianalves@ceb.uminho.pt](mailto:dianalves@ceb.uminho.pt)


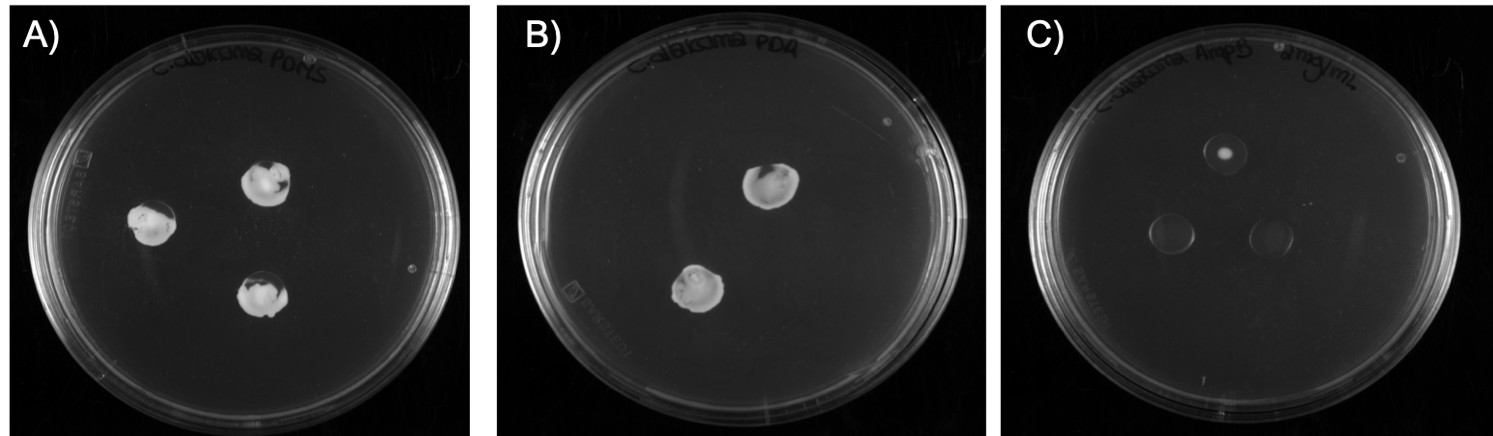


**Supplementary Figure 1.** Representative pictures of contact-killing assay. Fungal growth can be observed on SDA plates containing the PDMS samples (A) and pDA-coated coupons (B) and no significant visible growth was evidenced on pDA-coated PDMS surfaces further functionalized with LAmB (C) at 2 mg.mL^-1^.


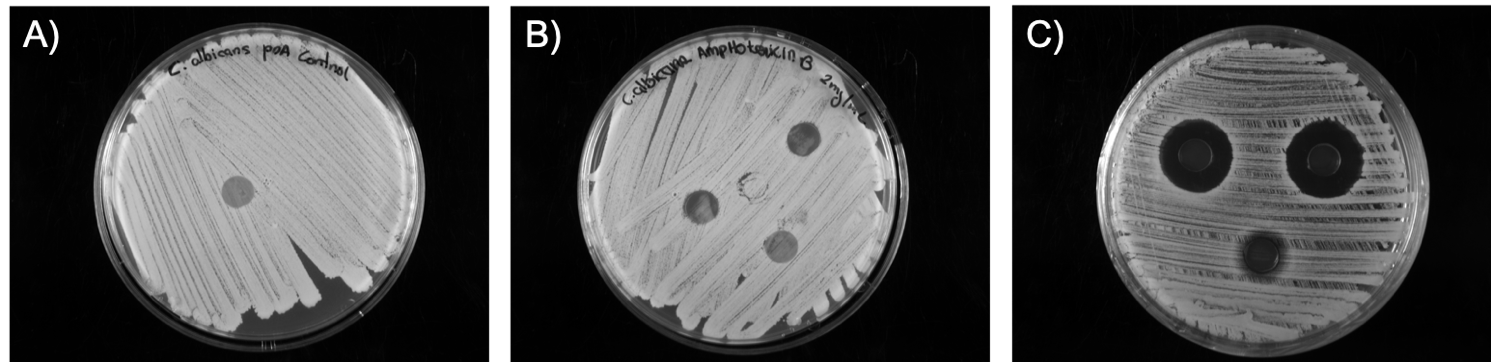


**Supplementary Figure 2.** Representative pictures of leaching assay. No evidence of inhibition zone was observed on pDA coupon (A) as well as on pDA-coated PDMS further functionalized with LAmB at 2 mg.mL^-1^ (B). On the other hand, the presence of an inhibition zone is evident around the surfaces functionalized with free AmB deoxycholate at the same concentration (C).
